# Totally Fluoroless Versus Limited Fluoroscopy-assisted Implantation: Identifying the Role of Fluoroscopy During Subcutaneous Implantable Cardioverter-defibrillator Implantation

**DOI:** 10.19102/icrm.2025.16101

**Published:** 2025-10-15

**Authors:** Stefanos Archontakis, Evangelos Oikonomou, Panagiotis Dourvas, Nikias Milaras, Damianos Kolios, Tzonatan Klogkeri, Epameinondas Triantafyllou, Christos Nikolaros, Anastasios Markakos, Artemis Papadima, Dimitra Tyrovola, Dimitrios Venetsanos, Dimitrios Sirseloudis, Sotirios Tsalamandris, Skevos Sideris

**Affiliations:** 1 Department of Cardiology, Hippokration General Hospital, Athens, Greece; 2 Third Cardiology Division, University of Athens, Medical School, Sotiria Thoracic Diseases Hospital, Athens, Greece; 3 Department of Cardiac Surgery, Hippokration General Hospital, Athens, Greece

**Keywords:** Fluoroscopy, implantable cardioverter-defibrillator, subcutaneous implantable cardioverter-defibrillator, sudden cardiac death, ventricular tachyarrhythmias

## Abstract

The subcutaneous implantable cardioverter-defibrillator (S-ICD) has emerged as an alternative to conventional ICD systems. Although not considered mandatory, short-time fluoroscopy is used in clinical practice, both preprocedurally and intraoperatively. The aim of this study was to compare totally fluoroless versus limited fluoroscopy-assisted S-ICD implantation in terms of clinical and technical efficacy and safety. In this non-randomized, single-center study, 49 patients (24.5% women; mean age, 43.2 ± 18.4 years) at high risk for arrhythmic cardiac death underwent S-ICD implantation in the context of either primary or secondary prevention between May 2016 and June 2024 with at least 6 months of follow-up thereafter. Patients were allocated to group A (n = 25), where a totally fluoroless implantation strategy was followed (January 2023–June 2024), or group B, where a limited fluoroscopy–guided S-ICD implantation process (first 24 cases) was followed. Following implantation, a pre-discharge chest X-ray confirmed an anatomically acceptable lead position in all cases. Further, our data revealed similar acute and long-term clinical efficacy with both approaches: the success rate of defibrillation testing at 60 J was 100%, the appropriate shock rate was low (8.2%) with defibrillation therapy successful in all cases, the mean PRAETORIAN score remained in the low-risk category, and no arrhythmic deaths were recorded. The rate of inappropriate shocks was similar between groups (8% vs. 8.3%; *P* = .97 for groups A and B, respectively). Finally, no major periprocedural complications were recorded with either approach. Compared to the limited fluoroscopy–guided technique, totally fluoroless S-ICD implantation showed comparable efficacy, reliability, and safety in the present study.

## Introduction

The subcutaneous implantable cardioverter-defibrillator (S-ICD) has emerged, in the last few years, as a promising alternative to conventional ICD systems, due to its ability to avoid potential periprocedural and long-term complications associated with the transvenous leads, such as pneumothorax, lead dislodgment and fracture, chronic lead failure, and systemic infections.^1–3^ Current evidence suggests that the S-ICD is highly effective and safe, with similar defibrillation success rates and low inappropriate shock rates compared to conventional ICDs.^4–13^

Despite these impressive achievements, the inability to perform anti-bradycardia pacing, in addition to technical difficulties in implantation, has significantly limited further outspread of this technology.^14,15^ Nevertheless, a wide range of patients who do not require pacing, particularly younger individuals, may benefit significantly.^15,16^ Currently, American Heart Association/American College of Cardiology/Heart Rhythm Society (AHA/ACC/HRS) and European Society of Cardiology (ESC) guidelines recommend the use of an S-ICD, with class I and IIa indications, respectively, in patients with an indication for ICD implantation and a high risk of infection or inadequate vascular access in cases where anti-bradycardia pacing, cardiac resynchronization therapy, or anti-tachycardia pacing (ATP) for ventricular arrhythmias is not considered necessary.^16,17^

In addition to omitting all risks related to transvenous lead implantation, fluoroscopy is not required during implantation of an S-ICD, as device positioning is guided by anatomical landmarks.^1,14,15^ Nevertheless, in clinical practice, most implanting physicians and several investigators recommend the use of short-time fluoroscopy both preprocedurally (to better specify the landmarks for operation and to maximize the left ventricular mass between the electrode and the pulse generator) and intraoperatively (to assess the position of the lead and generator in relation to the left ventricle), especially in patients who have undergone surgical thoracic operations, obese individuals, and patients with anatomical variations.^18–22^

The aim of this study was to carry out a comparative analysis in terms of clinical and technical efficacy and safety between totally fluoroless and limited fluoroscopy-assisted S-ICD implantation.

## Materials and methods

### Study design and patient population

This single-center, non-randomized, open-label study was performed in the cardiology department of our hospital between May 2016 and December 2024. All devices were implanted by a single operator experienced in placing implantable cardiac devices.

Patients who underwent S-ICD implantation were categorized into two groups, namely: (1) group A, where a totally fluoroless implantation strategy was followed, and (2) group B, where a limited fluoroscopy-guided S-ICD implantation process was used. Implantations in group A were performed between January 2023 and June 2024, and patients were followed up prospectively. Group A data were compared with those of group B, where implantations were performed between May 2016 and December 2022. In group B, patient data were retrospectively analyzed. Given the significant experience gained from the initial cases (ie, May 2016–December 2022), a transition in practice was made in January 2023, shifting from fluoroscopy-assisted implantation to a completely fluoroless technique.

S-ICDs were implanted in all patients as an alternative measure to transvenous ICDs (TV-ICDs) when pacing for bradycardia, cardiac resynchronization, or ATP was not considered necessary at the time of implantation or most probably in the future.^14^ In addition, an S-ICD was preferred compared to a transvenous system in cases of venous occlusion or thrombosis; in patients with a history of lead infection or previous extraction of a transvenous system; for implantation in immunocompromised individuals or patients in hemodialysis; and in patients with hypertrophic cardiomyopathy, primary electrical disease, and congenital heart disease, especially those of a young age.^14^ To ensure patient eligibility for an S-ICD, we performed an electrocardiographic (ECG) template screening in both groups to confirm a satisfactory R-wave/T-wave ratio pre-implantation in the lying and upright positions. In younger patients, a treadmill exercise stress test was performed prior to implantation. Patients with a prior sternotomy and those with a body mass index (BMI) between >3 (due to the higher risk of defibrillation failure) and <16 kg/m^2^ were excluded from the study.

Extensive and detailed information was provided to patients regarding the implantation process and the status of fluoroscopy during the S-ICD implantation process. All patients consented to allow access to their medical records for study purposes.

At baseline, demographic data, medical history, indication for a defibrillator system implantation, reason for S-ICD selection and/or TV-ICD rejection, and additional clinical data were recorded. Patients’ comorbidities, such as the presence of arterial hypertension, diabetes mellitus, dyslipidemia, chronic renal failure and/or hemodialysis, immunocompromised status, prior lead infection and/or extraction, established venous access problems, and atrial fibrillation, were systematically recorded.

Patients were admitted and discharged following one night’s stay after interrogation of the device, clinical assessment, and a chest X-ray. A scheduled visit at the device clinic was performed at 2 weeks, 3 and 6 months, and yearly thereafter. All patients had a follow-up period of at least 6 months. During each visit, the clinical status, presence of complications, and device parameters were recorded. Patients were also screened for inappropriate shocks, defined as any shock delivered for any condition other than ventricular tachycardia or ventricular fibrillation (VF).

The main endpoints of the study were as follows: (1) inability for effective defibrillation during testing; (2) failed defibrillation and/or episode of sudden arrhythmic cardiac death; (3) presence of major periprocedural complications, assessed during a hospital stay or the follow-up visits; and (4) presence of an anatomically non-acceptable lead position. In addition, the procedural time in all cases and fluoroscopy time for fluoroscopy-assisted procedures were recorded.

### Ethical considerations

This study was conducted in accordance with the Declaration of Helsinki and adhered to ethical guidelines for research involving human subjects. The study protocol was reviewed and approved by the institutional review board/ethics committee of Hippokration General Hospital, Athens, Greece. In the prospective study group (group A, January 2023–June 2024), all patients provided written informed consent before participation, having been fully informed about the implantation procedure, including the fluoroscopy status, and having consented to the collection, analysis, and publication of anonymized data. In the retrospective study group (group B, May 2016–December 2022), a waiver of ethical approval was granted by the ethics committee of Hippokration General Hospital, as the study involved only secondary use of anonymized patient data from medical records without direct patient contact or intervention. The retrospective analysis posed minimal risk to participants, and all data were handled in compliance with institutional and national data-protection regulations. All personal data stored in electronic and hard-copy forms were pseudonymized.

### Implantation technique

All procedures were performed in the electrophysiology laboratory under general anesthesia and continuous ECG and blood pressure monitoring in the presence of an anesthetist using the two-incision technique. Prophylactic antibiotics were administered.

The implantation technique has been described in detail elsewhere.^1,2,11^ Briefly, first, the optimal positions for the implantation of the electrode and the device were assessed, before preparation and draping, by means of anatomical landmarks, including (1) a 2-cm-long horizontal incision, lateral to the xiphoid, and (2) a 6-cm left lateral incision along Langer’s lines over the sixth rib in the anterior axillary line. The incisions as well as the generator’s outline were then marked on the skin. In addition, a demonstration electrode and a generator were used to further optimize the implantation positions. The lead was placed externally, 1–2 cm left lateral to the sternum, and the proximal ring was placed 1 cm superior to the xiphoid, whereas the demonstration generator was placed at the sixth intercostal space at the midaxillary line.

**Figure 1:**
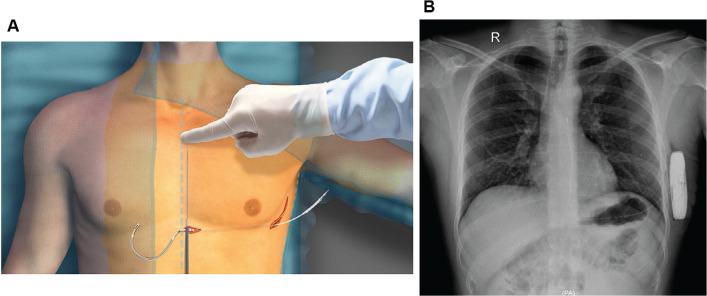
Subcutaneous implantable cardioverter-defibrillator implantation. **A:** Schematic presentation (used with permission from Boston Scientific). **B:** Post-implantation chest X-ray in a 42 year-old male patient with Brugada syndrome, with limited fluoroscopy assistance.

Next, following preparation of the skin and surgical draping, the device pocket was created beneath the latissimus dorsi muscle on the left, between the fifth and sixth intercostal spaces, near the midaxillary line. In addition, a 2-cm incision at the xiphoid level was made. A subcutaneous tunnel was then created by means of the electrode insertion tool (EIT), through which the electrode was drawn from the pocket to the xiphoid incision, where the electrode shaft was fixed using non-absorbable suture material. Then, the peel-away sheath was placed over the shaft of the EIT and tunneled in parallel to the sternal lateral edge approximately 1 cm to the left of the sternal midline for 14 cm superior to the xiphoid incision. Subsequently, the EIT was removed, leaving the peel-away sheath in a subcutaneous position. The electrode was then inserted into the subcutaneous sheath until the distal tip reached the opening of the sheath, and then the sheath was peeled away, leaving the electrode in place. During this process, the tip of the lead was guided by mild compression to reach the sternomanubrium site. Finally, the proximal end of the lead was connected to the device header, the generator was fixed into the subcutaneous pocket, and the skin was closed using standard suture technique **(Figure 1)**.

In group B, where limited fluoroscopy-guided implantation was performed, a few seconds of fluoroscopic assessment at the anteroposterior and lateral views was completed. More specifically, (1) a preprocedural short fluoroscopic assessment of the position of the demonstration lead and device was performed to increase the ventricular mass enclosed between the lead and the generator and to localize the position of the distal ring and (2) a short fluoroscopic assessment of the final position of the lead was performed at the end of the operation.

**Figure 2:**
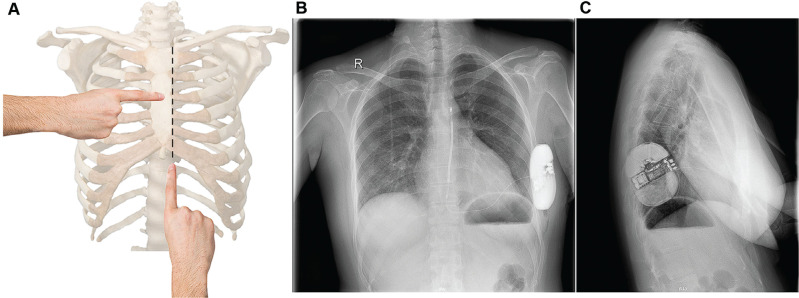
Totally fluoroless subcutaneous implantable cardioverter-defibrillator Implantation (S-ICD). **A:** Landmarks for totally fluoroless S-ICD Implantation. **B:** Post-implantation chest X-ray in a 26 year-old female patient with dilated cardiomyopathy.

On the other hand, among patients in group A, no fluoroscopic assessment was performed throughout the implantation process. Implantation was performed similarly to the technique previously described, by positioning the lead 1–2 cm lateral to the sternum. Initially, the xiphoid process was palpated and, subsequently, the left edge of the sternum was identified. While palpating the left edge of the sternum, the EIT was advanced cephalically **(Figure 2)**. The placement of the lead was solely guided by palpation without any fluoroscopic assistance.

### Periprocedural defibrillation threshold testing

Defibrillation testing (DFT) was performed, initially with 60 J and, if unsuccessful, subsequently with 80 J, after VF induction with 50-Hz stimulation. Polarity was automatically reversed in case of failure. The sensing vector was automatically selected by means of the setup algorithm.

### Programming

A dual zone was programmed in all patients with a minimum zone rate cut-off of 200 bpm. In addition, the SMART Pass™ algorithm (Boston Scientific, Marlborough, MA, USA) was activated in all cases.

### Statistical analysis

Continuous variables were expressed as mean ± standard deviation values if normally distributed and as median with interquartile ranges if not normally distributed. Normality was assessed through a visual inspection of the distribution and the Kolmogorov–Smirnov test. Comparisons between group A (totally fluoroless implantation strategy) and group B (limited fluoroscopy-guided implantation strategy) were performed using Student’s *t* test for normally distributed continuous variables and the Mann–Whitney *U* test for non-normally distributed continuous variables. Categorical variables were presented as absolute numbers and valid percentages. Comparisons between groups were performed using the chi-squared test or Fisher’s exact test, as appropriate, depending on expected cell counts. All statistical tests were two-tailed, with *P* < .05 considered statistically significant. Statistical analyses were performed using SPSS Statistics, version 29 (IBM Corp., Armonk, NY, USA).

## Results

We analyzed 49 patients subjected to S-ICD implantation between May 2016 and June 2023 who met the inclusion criteria. Of the evaluated patients, 12 (24.5%) were women and 37 (75.5%) were men. The mean age was 43.2 ± 18.4 years, and the mean BMI was 26.2 (range, 19.1–35) kg/m^2^. Patient baseline characteristics are summarized in **Table 1**. In nine (18.4%) patients, S-ICD implant was indicated for secondary prevention. The indications for S-ICD implant were lack of access in 57.1% (n = 28) of cases, young age in 34.7% (n = 17) of cases, and immunosuppression in 8.2% (n = 4) of cases. Forty-eight of 49 patients had at least 6 months of follow-up assessment, whereas the remaining patient underwent extraction of the subcutaneous system, 63 days after implantation, due to skin erosion close to the xiphoid process that resulted in system infection with a non-response to antibiotic therapy. Individuals in the limited fluoroscopy-guided S-ICD implantation group were younger (*P* = .08), and their implantations in the context of secondary prevention were significantly more frequent (25% vs. 12% in group A; *P* = .3). A male sex predominance was recorded in both groups. Otherwise, there were no statistically significant differences in baseline characteristics between groups, including underlying heart disease, indications for S-ICD implantation, left ventricular ejection fraction, BMI, prevalence rates of diabetes mellitus and renal disease, and QRS duration (98.8 ± 21.53 vs. 97.0 ± 15.45 ms; *P* = .74). Moreover, there was no difference in the number of passing vectors (1, 2, or 3) among groups (*P* = .73). The PRAETORIAN score was calculated in 13 patients in group A and 17 patients in group B as 49.8 (range, 30–180) and 35.4 (range, 30–150) points, respectively. Among the 17 patients in group B, 15 had a low risk of conversion failure (88.2%). On the other hand, among the 13 patients in group A, eight had a low risk (61.5%) of conversion failure according to the PRAETORIAN score.

**Table 1:** Patient Demographics and Clinical Characteristics.

	Total (n = 49)	Group A Totally Fluoroless Implantation Strategy (n = 25)	Group B Limited Fluoroscopy-guided S-ICD Implantation (n = 24)	*P*
Age (years)	43.2 ± 18.4	47.6 ± 19.8	38.5 ± 15.9	.08
Female sex	12 (24.5%)	5 (20.0%)	7 (29.2%)	.45
BMI (kg/m^2^)	26.16 ± 4.63	26.92 ± 5.27	25.40 ± 3.85	.26
QRS duration (ms)	97.9 ± 18.56	98.8 ± 21.53	97.0 ± 15.45	.74
Underlying heart disease				
ICM	14 (28.6%)	8 (32.0%)	6 (25%)	.26
DCM	9 (18.3%)	6 (24.0%)	3 (12.5%)	
ARVC/D	3 (6.1%)	0 (0.0%)	3 (12.5%)	
HCM	10 (20.4%)	7 (28.0%)	3 (12.5%)	
Brugada syndrome	9 (18.3%)	3 (12.0%)	6 (25%)	
LQTs	1 (2.0%)	0 (0.0%)	1 (4.2%)	
CHD	2 (4.1%)	1 (4.0%)	1 (4.2%)	
Muscular dystrophy	1 (2.0%)	0 (0.0%)	1 (4.2%)	
Indications				
Lack of access	28 (57.1%)	15 (60.0%)	13 (54.2%)	.98
Young age	17 (34.7%)	8 (32.0%)	9 (37.5%)	
Immunosuppression	4 (8.2%)	2 (8.0%)	2 (8.3%)	
Secondary prevention	9 (18.4%)	3 (12.0%)	6 (25.0%)	.30
LVEF (%)	47.3 ± 12.5	44.5 ± 11.0	50.1 ± 13.5	.12
NYHA class				
I	31 (63.3%)	15 (60.0%)	16 (66.7%)	.36
II	12 (24.5%)	8 (32.0%)	4 (16.7%)	
III	6 (12.2%)	2 (8.0%)	4 (16.7%)	
DM	9 (18.4%)	4 (16.0%)	5 (20.8%)	.66
Renal disease	5 (10.2%)	2 (8.0%)	3 (12.5%)	.60
AF	7 (14.3%)	4 (16.0%)	3 (12.5%)	.72
Passing vectors (mean)				
1	2 (4.1%)	1 (4.0%)	1 (4.0%)	.73
2	17 (34.7%)	10 (40.0%)	7 (29.2%)	
3	30 (61.2%)	14 (56.0%)	16 (66.7%)	
PRAETORIAN score (n)	37.5 (30.0–67.5)	45.0 (30.0–120.0)	30.0 (30.0–60.0)	.12
Low risk	23 (76.7%)	8 (61.5%)	15 (88.2%)	.22
Intermediate risk	3 (10.0%)	2 (15.4%)	1 (5.9%)	
High risk	4 (13.3%)	3 (23.1%)	1 (5.9%)	

*Abbreviations:* AF, atrial fibrillation; ARVC/D, arrhythmogenic right ventricular cardiomyopathy/dysplasia; BMI, body mass index; CHD, congenital heart disease; DCM, dilated cardiomyopathy; DM, diabetes mellitus; ICM, ischemic cardiomyopathy; LQT, long QT syndrome; LVEF, left ventricular ejection fraction; NYHA, New York Heart Association; S-ICD, subcutaneous implantable cardioverter-defibrillator. Categorical variables are presented as numbers and valid percentages. Continuous variables are presented as mean ± standard deviation values when normally distributed or median with interquartile range values when not normally distributed. For categorical variables, *P* values are based on the chi-squared test or Fisher’s exact tests as appropriate. For continuous variables, *P* values are based on a *t* test or the Mann–Whitney test as appropriate.

In all patients, the device was successfully implanted and intraoperative DFT at 60 J was performed. We planned to repeat the test by administrating 80 J if the first 60-J standard polarity shock was unsuccessful; however, a successful DFT at 60 J and standard polarity were recorded in all 49 patients. Procedure-related details are summarized in **Table 2**. The procedural times were comparable between groups. The mean fluoroscopy time was 11 s for group B and did not exceed 30 s in any case. The presence of an anatomically acceptable lead position was assessed by means of a chest X-ray performed the day after implantation before discharge. The lead position was considered to be optimal in all cases, and no repositioning was required.

**Table 2:** Patient Outcomes

	Total (n = 49)	Group A Totally Fluoroless Implantation Strategy (n = 25)	Group B Limited Fluoroscopy-guided S-ICD Implantation (n = 24)	*P*
DFT (60 J)	49 (100%)	25 (100%)	24 (100%)	1.0
Inappropriate anatomical positioning and repositioning	0 (0.0%)	0 (0.0%)	0 (0.0%)	1.0
Appropriate shocks (6-month follow-up)	5 (8.2%)	2 (8%)	3 (8.3%)	.97
Inappropriate shocks (6-month follow-up)	4 (8.2%)	2 (8%)	2 (8.3%)	.97
T-wave oversensing	1 (2.0%)	1 (4.0%)	0 (0.0%)	
Atrial fibrillation	2 (4.1%)	0 (0.0%)	2 (8.3%)	.26
Myopotentials	1 (2.0%)	1 (4.0%)	0 (0.0%)	
Death from any cause (6-month follow-up)	2 (4.1%)	1 (4.0%)	1 (4.2%)	.95
Sudden arrhythmic death	0 (0.0%)	0 (0.0%)	0 (0.0%)	
Non-arrhythmic cardiac death	2 (4.1%)	1 (4.0%)	1 (4.2%)	.97
Non-cardiac death	0 (0.0%)	0 (0.0%)	0 (0.0%)	
Complications (total)	7 (14.3%)	3 (12.0%)	4 (16.6%)	.64
Infections (total)	3 (6.1%)	2 (8.0%)	1 (4.2%)	.57
30-day complications				
Infection	2 (4.1%)	1 (4.0%)	1 (4.2%)	
Hematoma	4 (8.2%)	1 (4.0%)	3 (12.5%)	.55
6-month follow-up complications				
Infection	1 (2.0%)	1 (4.0%)	0 (0.0%)	.30
Procedural time (min)	64 ± 9	62 ± 9	66 ± 10	.14
Fluoroscopy time (min)	N/A	N/A	11 ± 4	

*Abbreviations:* DFT, defibrillation testing; N/A, not available; S-ICD, subcutaneous implantable cardioverter-defibrillator.

**Figure 3:**
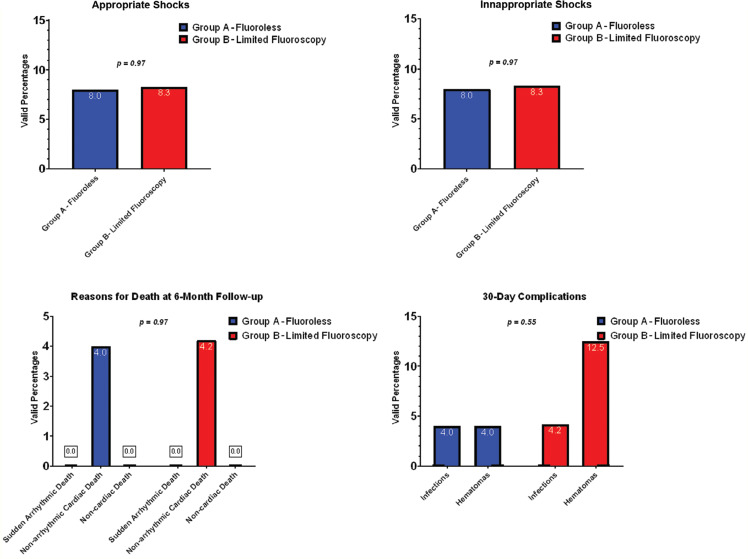
Comparative data between group A (fluoroless subcutaneous implantable cardioverter-defibrillator implantation) and group B (limited fluoroscopy-guided subcutaneous implantable cardioverter-defibrillator implantation). **A:** Inappropriate shocks. **B:** Appropriate shocks. **C:** Mortality within 90 days. **D:** Complications within 30 days.

Patient data were analyzed at 1-year post-implantation follow-up time with regard to appropriate and inappropriate shocks, hospitalizations, and the complication rate **(Table 2, Figure 3)**. In addition, death from any cause, arrhythmic deaths, and non-arrhythmic deaths were recorded. The rate of appropriate shocks was low, as five spontaneous episodes of VF occurred in four (8.2%) patients, and all episodes were successfully converted to sinus rhythm. Four inappropriate shocks occurred in four (8.2%) patients, and there was no significant difference in the prevalence between the totally fluoroless implantation group and the limited fluoroscopy-guided S-ICD implantation group (two shocks in each group). Two patients received an inappropriate shock due to atrial fibrillation paroxysm. One patient received a shock due to recorded myopotentials, whereas T-wave oversensing occurred in one patient. Overall, two deaths were recorded, one in each group, during the 6-month follow-up period. Both incidences were attributed to non-arrhythmic cardiac death due to pump failure. During this time period, no arrhythmic death was recorded. Additionally, we did not record any non-cardiac death.

The rate of complications in our patients during the first 30 days of follow-up was 12.2% (ie, six cases, including four cases of hematomas and two cases of localized infections). One case of infection and one case of local hematoma in group A and three cases of local hematomas and one case of infection successfully treated with antibiotics in group B were recorded, respectively **(Table 2)**. There was no electrode or pulse generator migration, and there was no need for a device reposition operation. A case of system infection occurred in a patient in group A, at day 62 after implantation, which eventually resulted in system extraction due to the lack of response to antibiotic therapy. No further complications were observed in group B beyond the first month of follow-up.

## Discussion

TV-ICDs remain the cornerstone for providing effective protection against life-threatening ventricular arrhythmias, both in the context of primary and secondary prevention of sudden cardiac death. However, despite their widespread application in clinical practice, the use of TV-ICDs is not without potential complications. The “Achilles heel” remains undoubtedly the transvenous lead, which is related to the most common complications such as pneumo-/hemothorax, vein thrombosis and occlusion, lead dislodgment, lead fracture, and lead failure with consequent pacing or sensing issues.^23^ In addition, infection, with an estimated incidence of 1.61%, remains the most feared complication related to cardiac device implantation.^24^

The S-ICD, introduced in clinical practice in the last 15 years, is a novel alternative that eliminates some risks associated with traditional TV-ICDs. In the S-ICD system, leads are entirely subcutaneous, resulting in a significantly lower rate of lead-related complications.^11,13^ Data from the ATLAS trial reported a 4.8% lead complication rate in the TV-ICD group compared to 0.6% in the S-ICD group at 6 months.^13^ In addition, the incidence of inappropriate shocks secondary to supraventricular arrhythmias is lower.^9,25^ The infection rate in patients with S-ICDs appears to be similar to that in patients with TV-ICDs^26^; nevertheless, most S-ICD infections are localized over the pocket or the lead trajectory in contrast to more life-threatening systemic infections and/or endocarditis usually occurring in patients with TV-ICDs requiring device extraction.^14^ Moreover, S-ICD infection responds adequately to conservative antibiotic treatment, and lead extraction, when required, is easier in patients with S-ICD systems compared to TV-ICD systems. On the other hand, S-ICDs are unable to perform pacing in bradycardic patients as well as ATP (DFT) in those with recurrent monomorphic ventricular tachycardia, and their implantation is associated with a greater incidence of inappropriate shocks secondary to T-wave oversensing.^26–28^ Currently, an S-ICD may be considered in younger patients, especially those with hypertrophic or dilated cardiomyopathy and channelopathies that usually do not require anti-bradycardia pacing or ATP, patients with congenital heart disease or difficulties in vascular access, and individuals with prior TV-ICD infections or other situations with a very high risk of infection of endovascular leads.^14,29^

Fluoroscopy is not considered necessary for S-ICD implantation. In this non-randomized, single-center study, 25 patients undergoing totally fluoroless S-ICD implantations were prospectively followed up for 6 months and data were compared, focusing on safety and clinical outcomes, with those of a retrospective control group of 24 patients in whom limited fluoroscopy-guided S-ICD implantation was performed. The major finding of the study is a similar acute and long-term clinical efficacy with both approaches **(Figure 3, Table 2)**. The success rate of the DFT at 60 J was 100% in both groups. In our patients, the appropriate shock rate was low (8.2%), and defibrillation therapy was successful in all cases, with no statistical differences between groups **(Figure 3, Table 2)**. Most importantly, no arrhythmic deaths were recorded. Of note, the PRAETORIAN score was higher in group A (with 61.5% of patients categorized as low risk) compared to group B (where 88.2% of patients had a low risk); however, the mean PRAETORIAN score remained in the low-risk category for conversion failure in both groups.

Another important finding is that the rate of inappropriate shocks, which was also low, showed no significant difference between the two groups (8% vs. 8.3% for groups A and B, respectively; *P* = not significant) **(Figure 3, Table 2)**.

Also, the absence of major periprocedural complications with either approach was recorded. Overall, the 30-day complication rate was higher in the fluoroscopy-assisted implantation group versus the totally fluoroless group (16.6% vs. 8%), reflecting gain of experience with the technique. Nevertheless, most complications were hematomas, with no clinically negative impact for the patients **(Figure 3)**. The device infection rate remained low in both groups A (n = 2) and B (n = 1); however, device extraction due to infection was required in a patient in group A with an infection that became evident 62 days postoperatively, without a response to antibiotics. None of the patients underwent an acute S-ICD reposition operation and lead positioning was optimal, as assessed by a chest X-ray prior to discharge. Finally, the procedural times were similar, and the total procedural fluoroscopy time in group B was <30 s in all cases.

Avoidance of fluoroscopy is considered to be a primary advantage of S-ICD implantation. In most of the early studies that provided solid evidence for S-ICD utility, anatomical landmarks instead of cardiac imaging were used during implantation.^1,4,30^ In the investigational device exemption study, fluoroscopy was used in only three (0.9%) cases for >1 min.^5^ Similarly, in clinical practice, fluoroscopy is not considered necessary during S-ICD implantation. Nevertheless, in practice, it is frequently used preprocedurally to assist with localization of the anatomical landmarks.^18,21,22,29,31^ During this procedure, once in the operating room, an S-ICD demonstration system, consisting of an S-ICD can and a coil, is positioned on the patient’s chest. The position of the device is assessed by fluoroscopy, and the demonstration system is subsequently repositioned in order to maximize the cardiac mass in the simulated defibrillation vector between the shock coil and the pulse generator. The pulse generator outline and the position of the sensing electrode and the distal sensing tip are then marked on the skin. Furthermore, some authors have used pre-implant screening guided by fluoroscopy in patients with an initially negative ECG screening result, who would otherwise be excluded to increase eligibility.^22^

During implantation, fluoroscopy is justified in patients with a prior sternotomy to prevent the S-ICD system coil from encountering the sternal wires.^32^ Moreover, in some centers, operators include in their practice the performance of a short (<30 s) fluoroscopy trial, intraoperatively, to assess the final position of the S-ICD system and avoid malpositioning.

Postoperatively, fluoroscopy has also been used in cases of failed defibrillation threshold testing to investigate whether the position of the electrode is suboptimal.^22,33^ Even after successful DFT, a short fluoroscopic test may be performed immediately postoperatively after the pocket is closed to demonstrate the assumed optimal position of the coil and to document an anatomically adequate defibrillation vector.^1^

In general, although fluoroscopy is not necessary and not recommended during S-ICD implantation, performing fluoroscopy for a few seconds, usually preoperatively and occasionally intra- or postoperatively to optimize the position of the implanted S-ICD system, has been adopted in the clinical practice in many implanting centers. A significant number of implanters consider a short fluoroscopy trial to be justified due to the associated non-significant radiation dose. On the other hand, currently, the effort to decrease radiation during interventional procedures to the lowest possible levels has become increasingly popular to omit any possible hazards for both the physician and the patient. In this context, the introduction of S-ICD into clinical practice contributes to the demand for a zero-fluoroscopy technique.

In the present study, we demonstrate that a limited fluoroscopy-guided S-ICD implantation approach does not yield additional benefits regarding DFT success, 1-year clinical defibrillation outcomes, or inappropriate shocks and carries a similar safety profile when compared to conventional fluoroless S-ICD implantation, and therefore it may be omitted. The fluoroless technique also prevents radiation exposure to patients and staff.

Nowadays, DFT is rarely performed during TV-ICD implantation. On the other hand, DFT of the S-ICD is still recommended at the time of implantation. Despite guideline recommendations, several implanting physicians omit DFT due to the possible complications as well as the high success rate of the test. The safety of not performing the DFT is still debated, however, as defibrillation success is influenced by several factors, such as implant position, device factors, and patient characteristics. Recently, it has been shown that a shock impedance of <90 Ω correlates with a DFT success rate of >95%.^34^ Therefore, it can be speculated that, in the presence of appropriate device parameters, such as a shock impedance of <90 Ω, both DFT and fluoroscopy may be omitted. However, additional studies are needed to justify this practice.

Our study should be interpreted in light of some limitations. First, although patients in group A were prospectively followed up with, the study design is retrospective and also non-randomized, and therefore selection bias cannot be excluded. Second, our study describes a single-center experience of the implementation of a totally fluoroless versus limited fluoroscopy-guided implantation, which may limit its generalizability. Third, time bias may have impacted our results, as totally fluoroless S-ICD implantation occurred later in time compared to limited fluoroscopy-guided implantation, and the operator performing totally fluoroless cases could have become more experienced with time. Fourth, a follow-up time longer than 6 months is probably needed to extract safer conclusions. Finally, all implantations were performed by the same operator. Moreover, fluoroscopy was used in the first period and its data were compared with experience from the second period, when this operator ceased using fluoroscopy, as the operator had gained significant experience from the first cases. The single-operator nature of the study as well as the difference in operator experience among the patient groups may have biased the results of this study.

## Conclusion

Avoidance of fluoroscopy during implantation remains a unique advantage of S-ICD. Compared to the limited fluoroscopy-guided technique, totally fluoroless S-ICD implantation showed comparable efficacy, reliability, and safety profiles in the present study. The S-ICD represents a valuable alternative to TV-ICDs, and our data support adaptation to a totally fluoroscopy-free approach as a standard of care due to increased patient and health care personnel safety with similar effectiveness and procedural safety profiles compared to the limited fluoroscopy-guided technique.
